# Individual Differences in *AMY1* Gene Copy Number, Salivary α-Amylase Levels, and the Perception of Oral Starch

**DOI:** 10.1371/journal.pone.0013352

**Published:** 2010-10-13

**Authors:** Abigail L. Mandel, Catherine Peyrot des Gachons, Kimberly L. Plank, Suzanne Alarcon, Paul A. S. Breslin

**Affiliations:** 1 Monell Chemical Senses Center, Philadelphia, Pennsylvania, United States of America; 2 Department of Nutritional Sciences, School of Environmental and Biological Sciences, Rutgers University, New Brunswick, New Jersey, United States of America; Erasmus University Medical Center, Netherlands

## Abstract

**Background:**

The digestion of dietary starch in humans is initiated by salivary α-amylase, an endo-enzyme that hydrolyzes starch into maltose, maltotriose and larger oligosaccharides. Salivary amylase accounts for 40 to 50% of protein in human saliva and rapidly alters the physical properties of starch. Importantly, the quantity and enzymatic activity of salivary amylase show significant individual variation. However, linking variation in salivary amylase levels with the oral perception of starch has proven difficult. Furthermore, the relationship between copy number variations (CNVs) in the *AMY1* gene, which influence salivary amylase levels, and starch viscosity perception has not been explored.

**Principal Findings:**

Here we demonstrate that saliva containing high levels of amylase has sufficient activity to rapidly hydrolyze a viscous starch solution *in vitro*. Furthermore, we show with time-intensity ratings, which track the digestion of starch during oral manipulation, that individuals with high amylase levels report faster and more significant decreases in perceived starch viscosity than people with low salivary amylase levels. Finally, we demonstrate that *AMY1* CNVs predict an individual's amount and activity of salivary amylase and thereby, ultimately determine their perceived rate of oral starch viscosity thinning.

**Conclusions:**

By linking genetic variation and its consequent salivary enzymatic differences to the perceptual sequellae of these variations, we show that *AMY1* copy number relates to salivary amylase concentration and enzymatic activity level, which, in turn, account for individual variation in the oral perception of starch viscosity. The profound individual differences in salivary amylase levels and salivary activity may contribute significantly to individual differences in dietary starch intake and, consequently, to overall nutritional status.

## Introduction

The initial digestion of dietary starch in humans is accomplished by salivary α-amylase, an endo-enzyme that catalyzes the hydrolysis of α-1,4 glycosidic linkages to produce maltose, maltotriose and larger oligosaccharides. This amylolytic digestion begins during mastication in the oral cavity, and continues within the stomach. The mixture then passes into the small intestine, where pancreatic amylase completes starch hydrolysis.

Salivary amylase is the most abundant protein in human saliva [1], accounting for 40 to 50% of salivary protein [2], and has the capacity to rapidly alter the physical properties of starch within the oral cavity [3]. The quantity and enzymatic activity of salivary amylase, however, show significant variation among individuals. This is due to a number of environmental factors, including stress levels [4], [5] and circadian rhythms [6]. In addition, there is evidence that salivary amylase expression is upregulated by a diet high in starch [7]. Genetically, salivary amylase levels are influenced by individual copy number variation (CNVs) of the *AMY1* gene on chromosome 1p21, which codes for salivary amylase [8]. The *AMY1* gene is one of the most variable CNV loci in the human genome, with a reported range of anywhere from 2 to 15 diploid copies. Importantly, oral salivary amylase concentrations positively correlate with the number of copies of the *AMY1* gene [9].

Genetic variation in *AMY1* appears to have evolved independently in diverse populations across the globe [9]. However, the nutritional advantage provided by the breakdown of starch in the oral cavity has never been established, since the majority of ingested starch is digested in the small intestine by pancreatic amylase. Two possibilities are that the presence of salivary amylase benefits nutrition 1) at the postprandial stage, by increasing the rate of blood glucose absorption from starch and 2) at the preprandial stage, by influencing the perception of textural attributes, such as viscosity, of starchy foods in the oral cavity. Perception of oral viscosity, or thickness, is a dynamic process that depends on the properties of the specific food being consumed, as well as changes in the food's structure that occur during oral manipulation [10]. These changes in viscosity play a significant role in determining liking and preference for a food. For example, the viscosity thinning of chocolate and ice cream in the mouth as they melt is considered central to their very high desirability and perceived creaminess [11], [12]. The degree to which the perceived viscosity of starch is thinned by the amylolytic “pre-digestion” of starch in the oral cavity is, therefore, nutritionally important.

Research using *in vitro* models to assess this relationship demonstrates that the enzymatic cleavage of starch produces a rapid decrease in glucose-polymer chain length and viscosity after relatively few glycosidic bonds have been cleaved [13]. However, *in vivo* research investigating the amylolytic decrease in viscosity in the oral cavity and its relevance to sensory perception has been difficult to observe and interpret. One study found that thickness ratings of starch-based custards were lower in subjects with high amylase activity in resting saliva but not in stimulated saliva [14]. However, since mastication preferentially increases output of the parotid gland (the main source of salivary amylase) [15], stimulated saliva would be expected to affect viscosity more than resting saliva. In another study, salivary amylase affected perceived viscosity only after amylase activity was modified by mixing custards either with additional amylase or the amylase inhibitor acarbose [16]. Linking oral amylase levels with perceptual sequellae may be complicated by the fact that individuals are accustomed to their own idiosyncratic salivary flow rates and amylase concentrations. Moreover, most studies of oral perception of starch have examined a single time point based on the assumption that amylolytic cleavage of glycosidic bonds occurs at a constant rate; however, *in vitro* rheological measurements demonstrate this is not the case [13].

Based on these considerations, we chose to examine how perceived viscosity changes in the oral cavity using time-intensity ratings, a method which more closely approximates the real-life perception of food changes over time. The goals of the current study were: 1) to determine how individual differences in salivary amylase levels affect starch viscosity breakdown using *in vitro* rheological measurements and 2) to elucidate how variation in salivary concentrations of the enzyme affects the perception of starch viscosity over time. Finally, despite the numerous environmental sources of variation in salivary amylase, we sought 3) to determine how polymorphisms of *AMY1* CNVs relate to salivary amylase concentration and enzymatic activity level, as well as to the perception of starch viscosity.

## Results

### Rheological Measures

**Figure 1:**
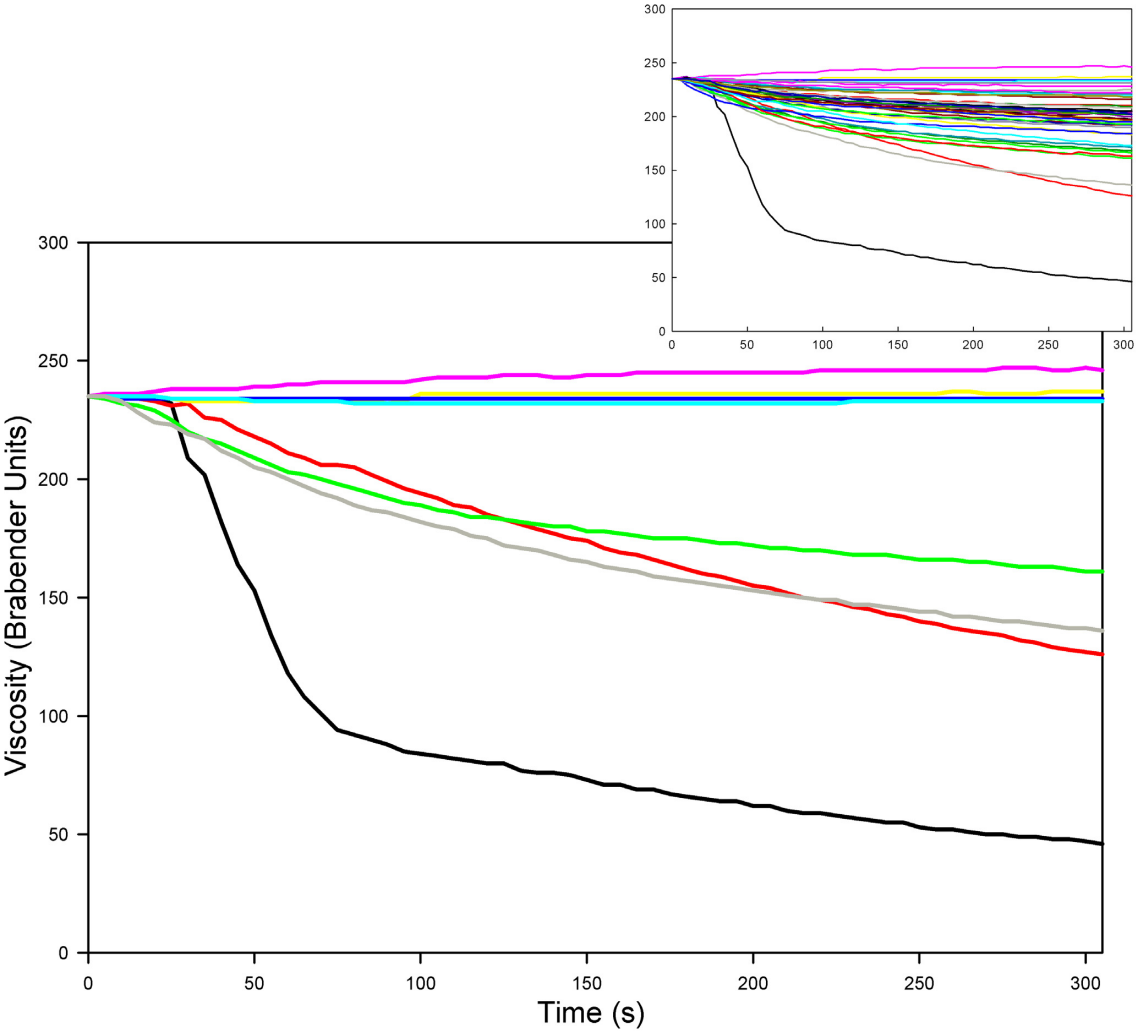
Starch viscosity measurements from the MVAG following the addition of subjects' saliva. This graph represents the four subjects with the least overall change in viscosity (upper curves) and the four with the greatest overall change (lower curves). The inset graph shows the data from all saliva samples analyzed in the MVAG (n = 42). In both graphs, the data from each subject is represented by a different colored line. 100 ul of each subject's saliva were added to 100 g of starch at 37.5°C. Saliva was added to the starch at time “0” and constituted ∼0.1% of the starch solution.

In the microviscoamylograph, the impact of saliva on starch viscosity varied between individuals from almost no impact to a rapid decline in starch viscosity within seconds. Figure 1 portrays the data from four subjects with the highest overall decreases in viscosity from 120 to 425 seconds and four subjects with the lowest changes. For all subjects, 100 ul of fresh saliva were added to 100 g of gelled 6% starch at time “0”. The viscosity decay curves overlapped for approximately 10 seconds for all individuals, indicating that amylase requires active mixing to become effective. After mixing, however, salivary amylolytic activity was highly individualized among subjects (See Table S1 in File S1). The inset in Figure 1 depicts the curves of all subjects to illustrate the full range of salivary activity.

### Salivary Amylase Measures

Immunoblotting and an enzymatic assay were used to independently quantify amylase amount/ml and activity/ml, respectively, in each saliva sample. We observed significant variation among individuals in terms of the amount and activity of amylase produced per unit saliva (Table S2 in File S1). The average amount (±SD) of amylase was 2.64 mg/ml (±1.8), with a range of 0 to 7.5 mg/ml, while the average concentration per minute was 5.7 mg/min (±7.1) (range 0–42.8 mg/min). The average activity per unit saliva was 93 U/ml (±62), ranging from 1 to 371 U/ml. The average activity per minute was 177 U/min (±166), with a range of 2 to 900 U/min. Males and females did not differ significantly in either their amylase amounts or activity.

**Figure 2:**
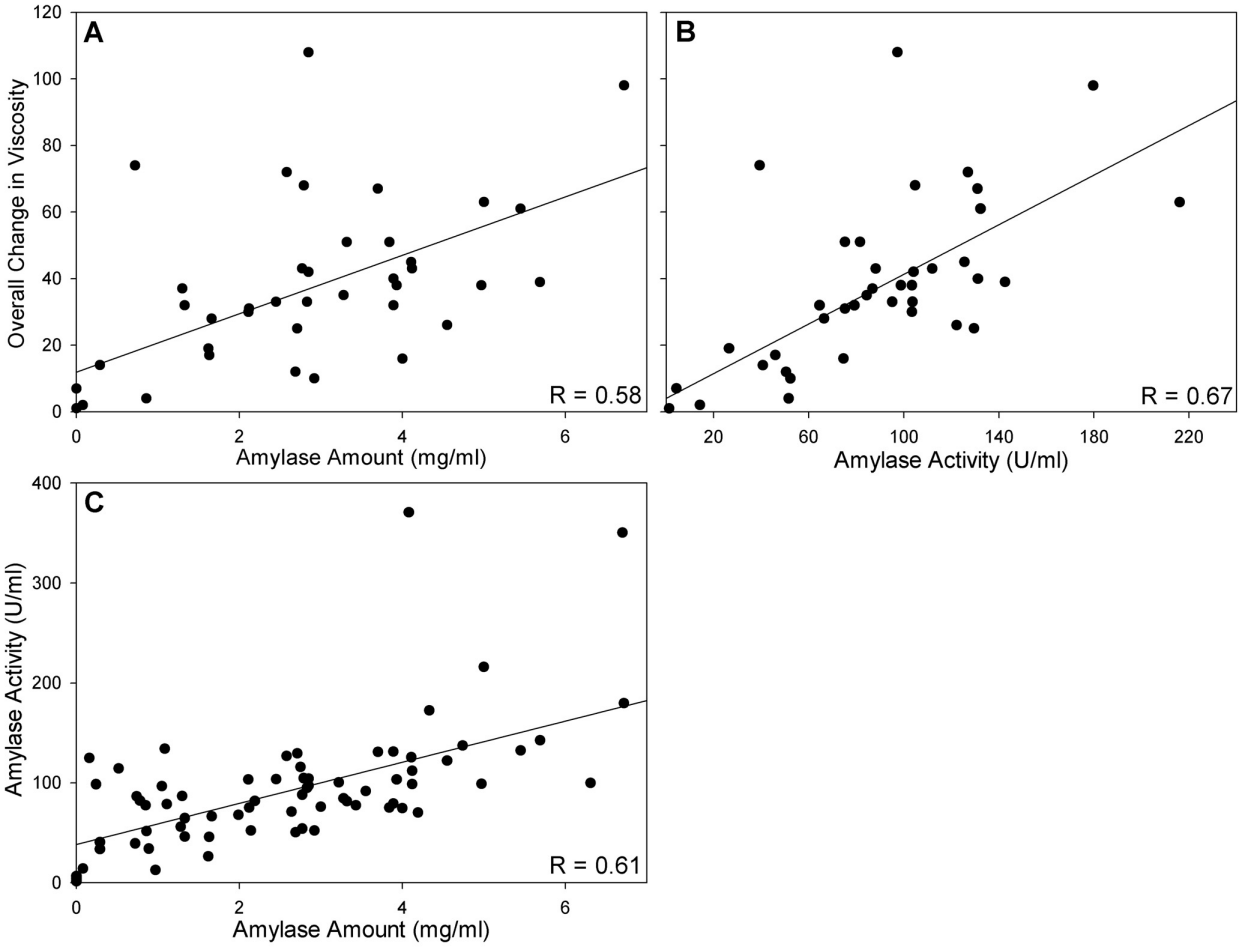
Correlations among salivary amylase measures. Both salivary amylase amount/ml (A) and salivary activity/ml (B) were significantly related to the overall change in viscosity measured by the MVAG. Salivary amylase amount/ml and salivary activity/ml were also significantly correlated with one another (C). Note that the saliva samples analyzed in the MVAG (n = 41) are a subset of those samples analyzed by Western blot and enzymatic assay (n = 73).

All three salivary measures (1. amylase amount per ml of saliva, 2. enzymatic activity per ml of saliva, 3. reduction of starch viscosity by 100 ul saliva injection into the MVAG) were significantly correlated with one another. The relationship between amylase amount (mg/ml) and overall viscosity change in the MVAG (Figure 2A) had an r value of 0.58 (P<0.0001) and the correlation between amylase activity (U/ml) and change in MVAG (Figure 2B) had an r value of 0.67 (P<0.001). As seen in Figure 2C, amylase amount and activity were significantly correlated with one another (r = 0.61; P<0.001), as well.

### *AMY1* Gene Copy Number and Salivary Amylase

**Figure 3:**
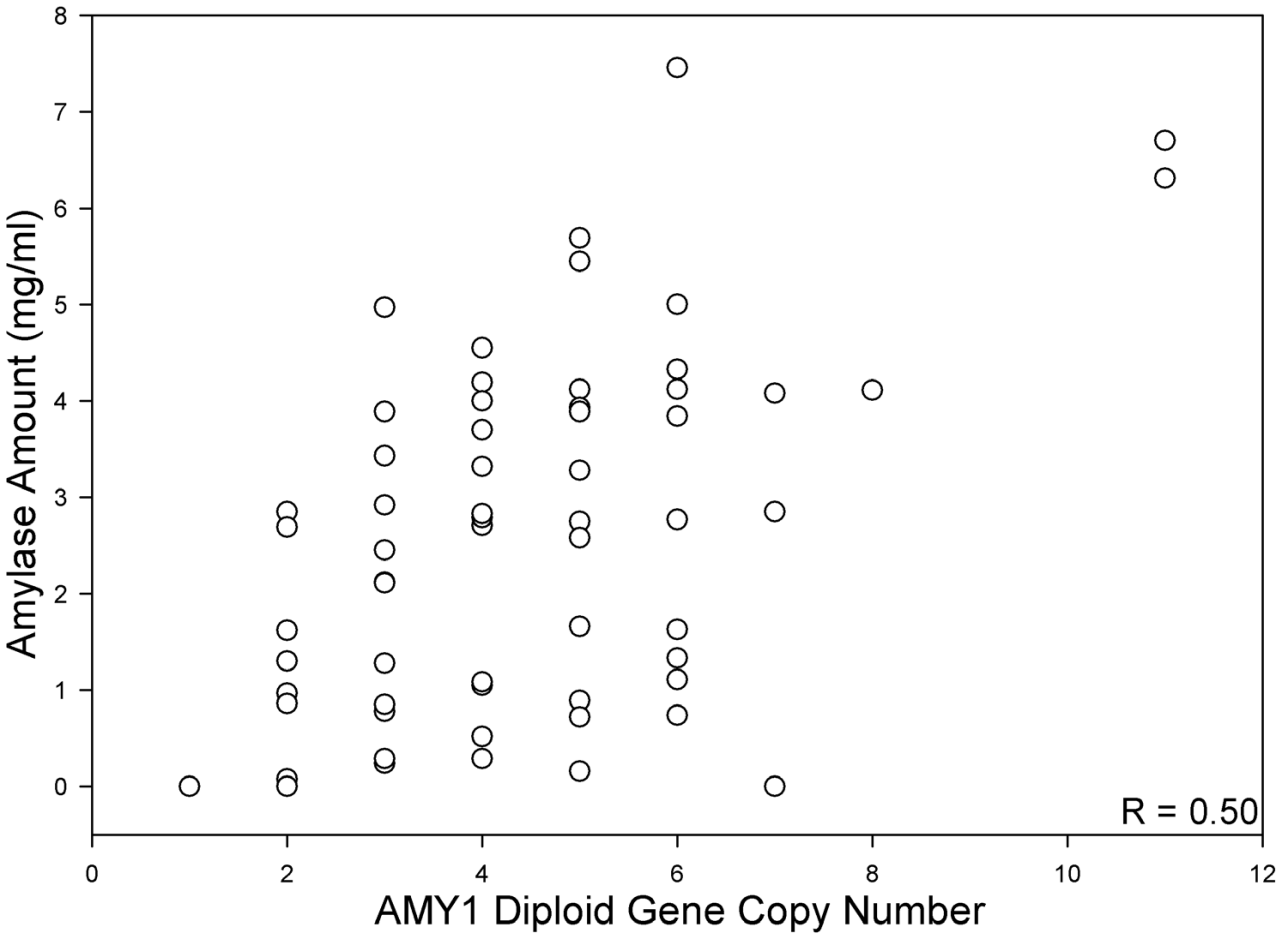
*AMY1* copy number variation and salivary amylase protein expression. There was a significant positive relationship between *AMY1* diploid copy number and amylase amount/ml (n = 62).

DNA samples were collected from 62 subjects and analyzed by qPCR to determine gene copy number. Values were standardized to a human DNA sample with a known *AMY1* gene copy number verified by Fiber FISH. The median number of *AMY1* gene copies was four (mean = 4.4±2), with a range of 1 to 11 (Table S2 in File S1). Salivary amylase amount/ml and gene copy number were significantly correlated (r = 0.50; P<0.0001; Figure 3). Salivary amylase activity/ml also increased as gene copy number increased (r = 0.52; P<0.0001) (not shown), consistent with the correlation between salivary amylase concentration and salivary enzyme activity (Figure 2C).

### Oral Perception of Viscosity

**Figure 4:**
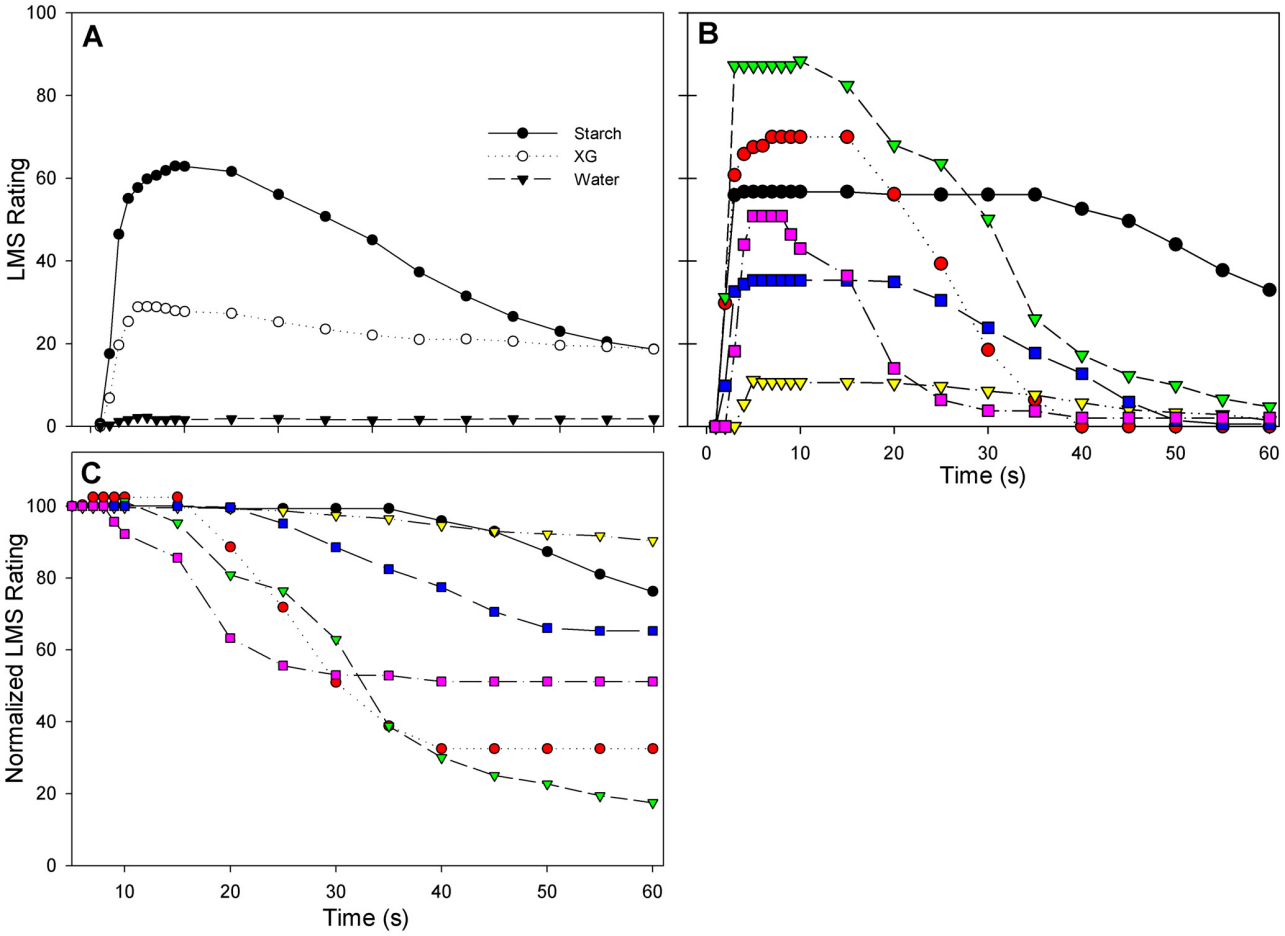
Subjects rated the viscosity of 6% starch, 1% xanthan gum, and water over the course of 60 seconds. Average time-intensity ratings for the three stimuli (A). As demonstrated by LMS ratings from six individuals (each portrayed by a different colored line/shape), subjects were highly variable in their use of the LMS scale when rating starch viscosity during the trial (B). LMS ratings were normalized to 100 at 5 seconds in order to remove subjective noise and enable observation of the effects of amylase on viscosity ratings (C). Note that Panels B and C contain LMS rating data from the same six subjects; each individual is represented by the same color line in each panel.

The mean perceived time-intensity viscosity functions of the three stimuli (starch, gum, and water) are presented in Figure 4A (See Table S3 in File S1 for data). As expected, subjects rated water as having a perceived viscosity very close to zero, which did not fluctuate during the 60 second measurement. After reaching a peak, ratings for the xanthan gum stimulus slightly decreased over the trial period, most likely due to volumetric thinning from salivary mixing, but otherwise remained stable over time. The shape of the starch viscosity rating curve suggested a two-stage process: an initial “mixing” phase, in which the subject manipulated the bolus in their mouth and mixed it with saliva (in Figure 4A, approximately 0–10 seconds) and a second “amylolytic activity” stage characterized by a negatively accelerating decrease in starch viscosity ratings over the remaining 50 seconds.

There were large individual differences in the viscosity ratings of starch (Figure 4B). To diminish the impact of subjective ratings, LMS ratings were normalized to 100, beginning at 5 seconds into the function (Figure 4C). The data were analyzed over the remaining 55 seconds by calculating 1) the overall change in ratings from peak to nadir and 2) the time at which the curve reached ½ viscosity rating following peak for each curve.

**Figure 5:**
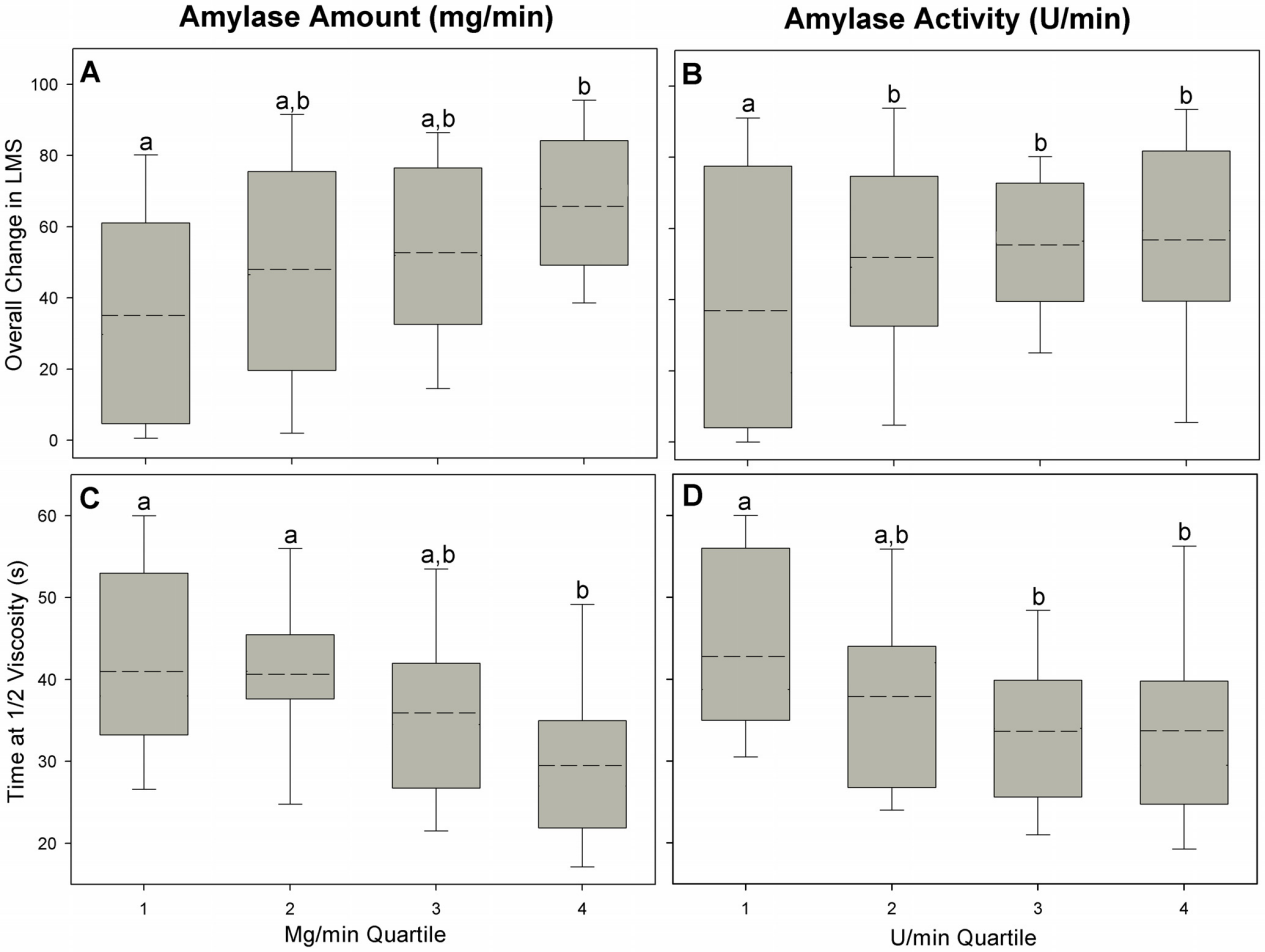
Salivary amylase levels were significantly related to perceived starch viscosity. Subjects with higher salivary amylase concentrations/ml (A) and salivary activity/ml (B) had greater overall changes in perceived viscosity. These subjects also reached ½ perceived viscosity levels significantly faster (C and D). The dashed line within each box represents the mean value, while the upper and lower boundaries of the box represent the 75^th^ and 25^th^ percentiles, respectively. The error bars above and below the box indicate the 90^th^ and 10^th^ percentiles. Points with different letters are significantly different from one another. Mg/min quartiles: 1 = 0–1.5; 2 = 1.51–2.99; 3 = 3–10; and 4 =  >10 mg/min. U/min quartiles: 1 = 0–60; 2 = 61–120; 3 = 121–220; and 4 =  >220 U/min.

In order to assess the relationship between the amount/activity of salivary amylase during the 60 second testing session and viscosity ratings, the *e*nzyme concentration/minute of saliva flow and activity/minute of flow were divided into quartiles. Subjects with higher salivary amylase concentrations (Figure 5A) (F(3,69) = 2.28, P<0.05) and salivary activity (Figure 5B) (F = 3.1, P<0.05) had greater overall changes in perceived starch viscosity than subjects with lower enzyme levels. Furthermore, these subjects also reported significantly faster decreases in viscosity over the course of the following 60 seconds (Figures 5C and D) (F = 3.12, P<0.05 and 3.2, P<0.05, respectively). Importantly, there was no significant relationship between overall change in the control stimulus (xanthan gum) viscosity and amylase levels (mg/min; P = 0.64) or activity (U/min; P = 0.51), which demonstrates the specificity of the enzyme for starch.

It is also worth noting that the *in vivo* LMS ratings of starch viscosity at 60 seconds were significantly related to the *in vitro* viscosity measurements from the MVAG at 7 minutes (r = 0.27; P<0.05). This highlights that the perception of starch viscosity as it breaks down in the mouth is directly related to the activity of salivary amylase on starch, since this is the only variable measured by the microviscoamylograph.

The relationship between *AMY1* gene copy number and the perception of starch viscosity was also examined. Overall change in perceived viscosity over time and the time to reach ½ perceived viscosity were not significantly related to the number of gene copies in this data set (P = 0.19 and P = 0.54, respectively) (not shown).

## Discussion

In the present study, we investigated the impact of individual differences in salivary amylase levels on the breakdown of starch viscosity *in vitro* and as perceived within the oral cavity *in vivo*. *In vitro* rheological measurements with a microviscoamylograph enabled direct observations of the rapid reduction in overall viscosity with very small volumes of fresh saliva (∼0.1% of the starch solution volume). Time-intensity ratings of perceived starch viscosity demonstrated the relevance of this amylolytic cleavage to oral sensory perception and its specificity for starch. Finally, we found that variation in *AMY1* gene copy number significantly influenced the concentration and activity of salivary amylase.

Our findings link variation in the *AMY1* gene to salivary amylase levels and activity *in vitro*, which, in turn, are related to the perceived breakdown of starch viscosity over time *in vivo*. Specifically, we observed that individuals with high levels of amylase experienced faster and more significant decreases in perceived starch viscosity than did individuals with low levels of amylase. Importantly, this method provides a more realistic assessment of viscosity perception than previous work, since individuals usually manipulate a starchy food in their mouth for several seconds (5 to 10 seconds depending on the food) to mix it with saliva before swallowing. Since barely any breakdown occurred in the xanthan gum condition and there was no relationship between XG viscosity change and amylase levels, we conclude that the changes in perceived viscosity of the starch stimulus were due to the action of amylase and not merely to salivary dilution. This is further supported by the relationship between the *in vitro* viscosity breakdown in the MVAG and the perceived viscosity changes over time *in vivo*.

In this study, individual differences in salivary amylase levels and activity were partially related to *AMY1* gene copy number. This finding corroborates the recent study of Perry and colleagues [9], which found that *AMY1* gene copy number is positively correlated with salivary amylase protein concentrations. Interestingly, Perry et al. also found that individuals from populations which historically consumed a high starch diet had significantly more copies of the *AMY1* gene than populations who subsisted on a protein-rich diet. Variation in copy number in the current study (average of 4.4 copies) is consistent with the observations of Perry et al. for those populations with low starch intake (5 copies) [9], suggesting that a significant proportion of our mixed-ancestry subjects may come from “low-starch” populations in which *AMY1* copy number is evolving more neutrally, rather than undergoing positive selection.

Whereas the specific fitness advantage conferred by upregulation of *AMY1* gene copy number and high levels of salivary amylase is unknown, it is possible that the enzyme activity affects preference and intake of starchy foods through its influence on the oral sensory properties of such foods. For example, salivary amylase levels affect both creaminess [16] and the release of flavor compounds [17] from starch-thickened custards; these characteristics are likely to affect an individual's liking of a food. Salivary amylase may also affect starch digestion and metabolism, as these factors are significantly affected by starch viscosity. Accordingly, when starch is delivered directly into the small intestine, skipping the oral “pre-digestion” by salivary amylase, significantly less digestion and glucose absorption occur [18]. This latter hypothesis suggests that individuals who produce high levels of salivary amylase and rapidly break starch into smaller glucose polymers may experience increased glycemic load from a high starch meal. Further research will be needed to test this hypothesis.

Based on the growing body of literature indicating that genetic variation can directly affect perception (taste, smell, etc), we also examined the relationship between *AMY1* gene copy number and oral viscosity perception. While we did not observe a direct relationship between these two sets of variables, this does not preclude the existence of such a relationship. Variation in gene copy number and in perceived viscosity are the most distal levels of analysis in this study, and many other variables may influence salivary amylase levels. For example, a significant amount of variation in protein amount and activity was not explained by *AMY1* gene copy number (R^2^ = 0.25 and 0.27, respectively) in this study. Our finding is consistent with previous research [8], [9], which suggests that amylase expression may reflect additional genetic influences, such as nonsynonymous single nucleotide polymorphisms (SNPs) in the *AMY1* gene (three known nonsynonymous SNPs) or differences in the transcription or translation efficiency between *AMY1* genes in different haplotypes [9]. Furthermore, nongenetic influences, including stress and dietary starch intake, may additionally affect protein levels. Regardless of the source of variability, recruitment of subjects with greater variation in gene copy number will be necessary to test this relationship further, since the majority (87%) of our subjects had between two and six copies of the *AMY1* gene.

In this study, we also examined the relationship between salivary amylase concentration and salivary enzymatic activity. Whereas the correlation between these measures was significant (r = 0.61; P<0.001), it is interesting to note that we did not observe a stronger relationship. It is possible to have two individuals with the same concentration of salivary amylase, with rather different levels of salivary enzymatic activity, and vice versa. This suggests that, for some individuals, amylolytic function of saliva may be affected by protein modifications (e.g. glycosylation) or the formation of complexes with other salivary proteins, such as mucins [19]. These factors, as well as the genetic influences mentioned above, may also explain the presence of outliers in this study; e.g. the two individuals in Figure 2C whose saliva exhibited much higher amylolytic activity than would be expected from the amount of amylase detected by immunoblotting.

One potential limitation of this study is our model oral bolus, parafilm, which, when chewed, may stimulate salivary flow rates that are only modestly comparable to those stimulated during oral manipulation of the starch bolus. We determined the amylase amount/min and activity/min from flow rates obtained by chewing parafilm, since making such determinations from an oral bolus of starch proved difficult. To this point, Mackie and Pangborn [20] demonstrated that amylase secretion rates (U/min) from the parotid gland while chewing parafilm and chewing bread did not differ, suggesting that parafilm is a reasonable model of an oral bolus.

This research demonstrates that salivary amylase plays a significant role in the oral perception of starch viscosity when saliva is mixed into a food. Salivary amylase levels are under both environmental and genetic controls. Understanding the factors that underlie our perception of starchy foods will help us to learn how the changes that occur in such foods during oral manipulation impact our liking, preference, and ingestion of such foods. The profound individual differences in salivary amylase levels and salivary activity, which are determined in part by our *AMY1* gene copy numbers, may contribute significantly to individual differences in dietary starch intake and, consequently, to our overall nutritional status. Future research will examine whether differences in oral amylase levels directly impact our liking for and consumption of starchy foods.

## Materials and Methods

### Ethics Statement

This study was approved by the Institutional Review Board for Human Participants at the University of Pennsylvania. All participants gave written informed consent before participating in the study.

### Subjects

Eighty-one healthy volunteers, ranging in age from 18–68 years, were recruited from the Philadelphia area and paid to participate in the study. Subjects were asked to refrain from eating or drinking anything other than water for one hour before sample collection.

Six subjects were excluded from the study based on their xanthan gum ratings data. Two additional subjects were removed due to the excessive amount of time they waited to begin rating the viscosity of the starch stimulus (≥20 seconds). The remaining 73 subjects (45 F, 28 M) had a mean age (± SD) of 29.5±8 years (age range: 18–68y). A subset of 42 subjects had their salivary activity measured in the MVAG. Sixty-two subjects donated DNA for CNV analysis.

### Saliva and DNA Collection

For the collection of stimulated, whole saliva, subjects chewed on a 4 cm square of parafilm to the beat of a metronome (80 beats/min) for 90 seconds and then expectorated into a 15 ml polypropylene tube. The tube was weighed before and after sample collection in order to calculate salivary flow rate (ml/min). The tube was vortexed and a small aliquot removed for use in the microviscoamylograph (see below). The tubes were then centrifuged at 2000× g at 4°C for five minutes and the remaining saliva was aliquotted and frozen at −20°C for future analysis. Upon thawing, the samples were centrifuged once more to ensure that solids were removed from suspension.

For genotyping, approximately 35 ml of blood were collected from each subject into a tube coated with EDTA to prevent coagulation. The tubes were inverted gently 10 times and then frozen at −20°C for future use. For subjects unable or unwilling to provide a blood sample (n = 25), cheek cells were collected using sterile foam-tipped applicators (Epicentre Biotechnologies, Madison, WI).

### Perceptual Testing

#### Stimuli

Subjects rated the viscosity of: a.) deionized water, b.) 1% xanthan gum (XG; ADM, Decatur, IL) homogenized with a dispersing agent, 0.025% NaCl, and c.) 6% gelatinized phosphate cross-linked waxy maize starch.

The water was used as a low viscosity standard stimulus. The xanthan gum (XG) was a viscosity control stimulus, since it can be solubilized to have a viscosity level comparable to that of 6% starch but is unaffected when mixed with salivary amylase.

The gelatinized starch was prepared by mixing the starch with purified water and heating in a 90°C water bath for 20 minutes. To ensure dispersion of the starch while heating, the beakers were removed from the bath and stirred magnetically every 3 minutes for 30 seconds. Samples were allowed to cool to room temperature before using.

#### Exclusion Criteria: Quality of Subject Ratings

In order to assess the ability to discriminate between different viscosities, subjects completed two tasks following saliva collection: a two alternative forced-choice (2-AFC) test and a viscosity acuity test. In the first task, subjects had to select the more viscous sample from cups containing 0.25, 0.50, or 0.75% XG. Subjects judged each randomly-presented combination five times, for a total of 15 presentations. In the latter test, subjects rated the viscosity of 0.25, 0.50, 0.75, 1, and 2% XG samples using a computerized labeled magnitude scale (LMS) system. Stimuli were presented in random order and each XG concentration was presented in duplicate.

The exclusion criteria were based on an overall score that combined a subject's performance on the forced-choice task and the viscosity ratings as follows: ¼*AFC correct score +¾*slope of LMS viscosity ratings function. Subjects were removed from further data analysis if they were unable to attain an overall score of 18.75 *and* they had greater than one rating inconsistency as XG viscosity increased. An inconsistency was counted when a subject rated one XG concentration as less viscous than a lower XG concentration (e.g. rating 0.75% XG as less viscous than 0.25% XG). This procedure ensured that people not able to perform the task properly were removed from the study.

#### Time-Intensity Labeled Magnitude Scale

Before beginning formal perceptual testing, subjects were asked to rinse their mouth three times with deionized water. They were presented with randomly ordered small medicine cups containing 10 ml of water, 1% XG, or 6% starch. Subjects rated the viscosity of each solution using a computerized LMS, which ranged from “zero viscosity” (i.e. water) to “most viscous” (i.e. a solid) with semi-logarithmically placed labels [21]. Subjects were instructed to manipulate each sample throughout their entire mouth, but not swallow, and continuously rate the viscosity for 60 seconds using the computer mouse. Viscosity rating data were automatically collected by the computer every second. The oral sample was then expectorated. Subjects rinsed their mouth three times with water between each trial. Subjects rated the viscosity of the three stimuli once, with a 5 second interval in between each stimulus. A subset of subjects (n = 33) were tested in two different sessions to assess reproducibility of ratings. LMS ratings were reproducible (r = 0.61, P<0.001).

### Rheological Measurements

The effect of each subject's freshly collected saliva on the viscosity of the starch solution was determined using a microviscoamylograph (MVAG; Brabender, Duisburg, Germany). This method enabled us to observe the degree of amylolytic activity present in each saliva sample. The viscosity of 100 g of gelatinized starch at 150 rpm and 37.5°C was automatically recorded by computer every 5 seconds for 2 minutes to establish a stable baseline viscosity measurement. After two minutes, 100 ul of centrifuged saliva were added via the injection port during rotation. The added saliva constituted approximately 0.1% of the total starch solution in the MVAG. Viscosity measurements were recorded for an additional 5 minutes. Controls trials, in which purified water was substituted for saliva, were also run.

### SDS-PAGE and Immunoblotting for Salivary Amylase

In order to quantify the amount of salivary amylase in each sample, 0.5 ul of saliva were assayed under reducing conditions and resolved by SDS-PAGE on a 4–15% gradient gel. Several different concentrations of commercially obtained human salivary α-amylase (0.9 to 15.1 ug; Sigma-Aldrich, St. Louis, MO) were also assayed to generate a standard curve for spot densitometry. Proteins were transferred onto a PVDF membrane (Millipore Corp, Bedford, MA) in transfer buffer for 1 hour at 300 mA. Membranes were blocked for 30 minutes at room temperature in blocking buffer (PBS, 0.1% tween) with 5% BSA. Membranes were incubated for 1 hour at room temperature with rabbit anti-α-amylase (Sigma-Aldrich), diluted 1∶7,500 in blocking buffer with 1% BSA. After washing in PBST, membranes were incubated for 45 minutes in donkey anti-rabbit IgG-HRP conjugate (GE Healthcare, Piscataway, NJ), diluted 1∶15,000 in blocking buffer with 1% BSA. Membranes were washed again and an ECL Plus Chemiluminescence system (GE Healthcare) was used for detection of amylase. Spot densitometry for the standards and samples was performed using the Alpha Imager software program (Alpha Innotech Corp, San Leandro, CA). Amylase concentrations in the samples were determined using the generated standard curve.

### Enzymatic Activity Assay for Salivary Amylase

Salivary amylase activity was determined using a kinetic reaction assay kit (Salimetrics LLC, State College, PA). This method measures amylolytic activity using a chromagenic substrate, 2-chloro-p-nitrophenol, linked to maltotriose. Amylase activity was measured according to the manufacturer's protocol, with the following modifications: 1) the assay was run at room temperature with heated substrate and 2) the optical density of the samples was read on a spectrophotometer at 405 nm at 2 and 5 minutes. Under these conditions, the intra-assay variation (CV) calculated from 11 replicates was 5.7%, while the inter-assay variation of 12 separate runs was 7.2%. The same saliva aliquot was used for both the Western blot (above) and enzymatic activity assay described here.

### Quantitative Polymerase Chain Reaction (qPCR) for the AMY1 Gene

In order to determine diploid *AMY1* gene copy number, DNA was extracted from whole blood using the Gentra PureGene DNA extraction kit (Qiagen, Valencia, CA). For those subjects who provided cheek cells, DNA was extracted using QuickExtract DNA extraction solution (EpiCentre). Extracted DNA was quantitated using a NanoDrop (Thermo Scientific, Wilmington, DE) and all samples were standardized to 5 ng/ul. A TaqMan Copy Number Assay for *AMY1* (Assay ID Hs07226362_cn) and RNAse P reference assay (44003328, Applied Biosystems, Foster City, CA) were used with TaqMan Genotyping Master Mix (Applied Biosystems), according to product literature. Twenty ul reactions (final DNA concentration of 1 ng/ul) were run in triplicate on an ABI Prism 7000 (Applied Biosystems). Data was analyzed using Copy Caller Software (Applied Biosystems). *AMY1* diploid copy number was estimated using a standard curve constructed from a reference DNA sample (NA18972; Coriell Cell Repositories, Camden, NJ), previously determined to have 14 *AMY1* diploid copies by qPCR and Fiber FISH [9].

### Statistical Analysis

Statistical analyses were performed using Statistica 8.0 software (Statsoft, Inc, Tulsa, OK). Relationships between data sets were analyzed using the Pearson correlation coefficient.The relationship between viscosity perception and salivary amylase measures were analyzed using one-way ANOVAs, with amylase quartiles as the categorical factor. The effects of *AMY1* gene copy number on viscosity perception were also analyzed using a one-way ANOVA, with copy number as the categorical factor. A P value (two-tailed) of <0.05 was considered significant.

## Supporting Information

File S1 Raw data for MVAG, amylase and perceptual ratings.
(0.14 MB XLS)
